# How Well Can Modern Nonhabitual Barefoot Youth Adapt to Barefoot and Minimalist Barefoot Technology Shoe Walking, in regard to Gait Symmetry

**DOI:** 10.1155/2017/4316821

**Published:** 2017-10-29

**Authors:** Y. Xu, Q. Hou, C. Wang, T. Simpson, B. Bennett, S. Russell

**Affiliations:** ^1^Department of Rehabilitation Medicine, The 1st Affiliated Hospital, Sun Yat-sen University, Guangzhou 510080, China; ^2^Motion Analysis & Motor Performance Laboratory, Department of Orthopedics and Mechanical Engineering, University of Virginia, Charlottesville, VA 22903, USA; ^3^Department of Neurology, The Seventh Affiliated Hospital, Sun Yat-sen University, Shenzhen 518107, China; ^4^Department of Kinesiology, California State University East Bay, Hayward, CA, USA

## Abstract

We aim to test how well modern nonhabitual barefoot people can adapt to barefoot and Minimalist Bare Foot Technology (MBFT) shoes, in regard to gait symmetry. 28 healthy university students (22 females/6 males) were recruited to walk on a 10-meter walkway randomly on barefoot, in MBFT shoes, and in neutral running shoes at their comfortable walking speed. Kinetic and kinematic data were collected using an 8-camera motion capture system. Data of joint angles, joint forces, and joint moments were extracted to compute a consecutive symmetry index. Compared to walking in neutral running shoes, walking barefoot led to worse symmetry of the following: ankle joint force in sagittal plane, knee joint moment in transverse plane, and ankle joint moment in frontal plane, while improving the symmetry of joint angle in sagittal plane at ankle joints and global (hip-knee-ankle) level. Walking in MBFT shoes had intermediate gait symmetry performance as compared to walking barefoot/walking in neutral running shoes. We conclude that modern nonhabitual barefoot adults will lose some gait symmetry in joint force/moment if they switch to barefoot walking without fitting in; MBFT shoe might be an ideal compromise for healthy youth as regards gait symmetry in walking.

## 1. Introduction

Currently, there has been a renewed enthusiasm for barefoot running. Yet, kinetics and/or kinematic observations on modern people walking or running in Minimalist Bare Foot Technology (MBFT) shoes or barefoot are not conclusive. For example, Sinclair [1] conducted a 3D running analysis in 30 recreational male runners and found that though barefoot and MBFT footwear significantly reduced patellofemoral kinetic parameters at the knee, they significantly increased Achilles tendon force at the ankle as compared to conventional shoes. And in 14 male rear foot striking runners, when they first took on MBFT shoes and ran on a treadmill, Willy and Davis [2] noticed that they had increased, rather than decreased, load on lower extremities as compared to their standard shoe running. And these kinetics and kinematic disadvantages of barefoot and MBFT footwear, have been noticed to be associated with overuse injuries or greater risk of fractures by several previous studies [3, 4].

Gait symmetry has received less attention as compared to other kinetic or kinematic variables in footwear studies, as there was a preconceived recognition that nondisabled healthy adults should naturally have symmetry gait. Nonetheless, accumulating evidences now indicate that it might not be the case in the general population, for example, by pooling bilateral gait data of 182 healthy subjects and using a clinically relevant asymmetry with a cut-off value of 10% between limbs. Lathrop-Lambach et al. [5] reported that more than half of the tested subjects manifested asymmetry in peak hip and knee flexion and adduction moments. Using a self-developed relative asymmetry index, Forczek and Staszkiewicz [6] noticed in 54 normal adults (27 women and 27 men) that asymmetry in joint angle in the sagittal plane was found in ankle joint despite they were strictly screened for limb length discrepancy (1 cm or more were excluded), suggesting that human walk is not perfectly symmetrical even in a group of relatively homogenous people. It is now increasingly recognized that asymmetric gait would undermine the alignment and configuration of lower limb joints and hence contribute to the pathologies and a risk of injury in the lower extremities [7–10].

This leads us to a question: how well will nonhabitual barefoot modern youth adapt to barefoot or MBFT shoe walking in the perspective of gait symmetry after having habituated to different style of shoes? Using a recently developed consecutive symmetry index (SI) that is able to quantify lower extremity symmetry either categorically for the motion in each of the three motion planes and at each of the three lower limb joints or globally for the motion in all the three motion planes or at all the three lower limb joints [11], we aim to evaluate gait asymmetry performance in a group of healthy university students for 3 walking conditions: barefoot, in MBFT shoes, and in neutral shoes. We hypothesis that having developed walking patterns with shoes, modern nonhabitual barefoot youth might need to readapt to barefoot or barefoot mimic walking conditions, in the perspective of gait symmetry.

## 2. Materials and Methods

30 university students were recruited through convenience sampling for the study. All the participants were healthy and free of diabetes mellitus, orthopedics, and neuromuscular diseases, and with no obvious structural asymmetry of bilateral lower limbs. The study was conducted in the Motion Analysis and Motor Performance Laboratory at the University of Virginia (UVA). The Human Investigation Committee of UVA monitored and approved all procedures of the present study. Consent was obtained for each participant enrolled (HSR#:16853).

**Figure 1:**
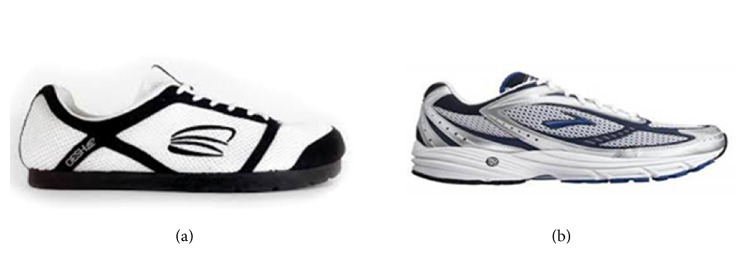
Two types of footwear tested: MBFT shoe (a) and neutral shoe (b).

The study protocol consisted of 3-dimension (3D) gait analysis for 3 walking conditions: barefoot, in MBFT shoes, and in neutral shoes (Figure 1). The OESH® (La Vida, from the OESH Barefoot Technology®) shoes that have completely flat soles, with no arch support and no heel lift, which aim to mimic barefoot, were used as the MBFT shoe condition in this study. A current widely used neutral running shoe (Brooks®, Radius 06) was used as the neutral shoe condition. The order of walking conditions tested was randomly decided by coin flipping of each enrolled individual.

Enrolled subjects were instructed to walk along a 10-meter laboratory walkway at their self-selected comfortable walking speed (CWS), wearing a Plug-in-Gait full body 37-marker set (Vicon, Oxford, UK). 3D kinematic and kinematic data were collected with an 8-camera Vicon Motion Analysis System (Vicon, Oxford, UK) at 120 Hz, and the data of contact reaction forces was collected using 4 in-ground force plates (Kistler, Switzerland and Betec, OH) at 1080 Hz. The acceleration and deceleration at the force plate were well controlled in each trial, and trials with walking speeds close to barefoot walking tests were chosen from the OESH shoe and neutral shoe walking tests; at least 5 successful trials which met these criteria were recorded for each subject.

Gait kinetics and kinematics variables were computed using Vicon's full body Plug-in-Gait models [12]. All variables were normalized to the stance phase. To evaluate gait symmetry, the equations for symmetry index (SI) below developed by Nigg et al. [11] were applied:

(1) SI=∫t=t1t2AXrt−Xltdt,

(2) A=2rangeXrt+rangeXlt,

where *Xr*(*t*) and *Xl*(*t*) are specific variables recorded for the right leg or the left leg at the time *t* and *t*1 and *t*2 refer to the times at heel-contact and toe-off, respectively. Therefore, evaluating gait symmetry with this SI consecutively incorporates the data of entire stance phase rather than discrete time points. In (1), *A* is used to normalize the data over range, and range is used instead of the mean of the data so that nonsimilar gait parameters could be compared. For any given variable, SI = 0 means perfect symmetry, while on the contrary, the larger the SI value is, the less symmetric the gait it indicates.

As per previous research, the variables of joint angle, joint force, and joint moment [5, 13] were selected for the calculation of SI, using the software of MATLAB® (The MathWorks, Inc., Natick, MA). According to Nigg's methodology [11], SI was calculated separately for each of the three joints of the lower limb (hip/knee/ankle) and for each of the three motion planes (sagittal/transverse/frontal) and was also calculated jointly with data from all the 3 lower limb joints and all the 3 motion planes, so that gait symmetry can be evaluated from both categorical and global perspectives.

All statistics analysis was performed with SPSS 20.0 for windows (SPSS, Inc., Chicago, IL). Continuous variables with normal distribution were presented as mean ± standard deviation. Leven's test was conducted to test the heterogeneity of data and log-transformed the data if necessary. One-way ANOVA was used for the intergroup comparisons; nonparametric tests were applied if heterogeneity of the data was not fulfilled by log transformation. *p* < 0.05 was considered as statistically significant.

## 3. Results

Two subjects failed to complete the whole testing procedure and were excluded from the final analysis. Thus, all utilized data was from 22 female and 6 male tested subjects (age: 20.14 ± 0.76 years; mass: 64.18 ± 9.03 Kg; height: 167.45 ± 5.81 cm; BMI: 22.98 ± 3.71 kg/m^2^; right handedness: 20; left handedness: 8.).

As expected, enrolled subjects walked faster on average with shoes than walked barefoot (walking speed (m/s): in MBFT shoe: 1.29 ± 0.11; in neutral shoe: 1.32 ± 0.13; barefoot: 1.23 ± 0.11; the difference between walking in neutral shoe and walking barefoot was significant on statistic, *p* < 0.05). To make walking speed comparable for kinematic and kinetic variables analysis, only trials with walking speeds close to that of the barefoot walking in the MBFT shoe and neutral shoe walking tests were included for analysis. After this adjustment, walking speed was not significantly different between each pair of the 3 conditions. Others spatiotemporal parameters, for example, step length, step cadence, and stride length, were all intergroup comparable as well.

**Figure 2:**
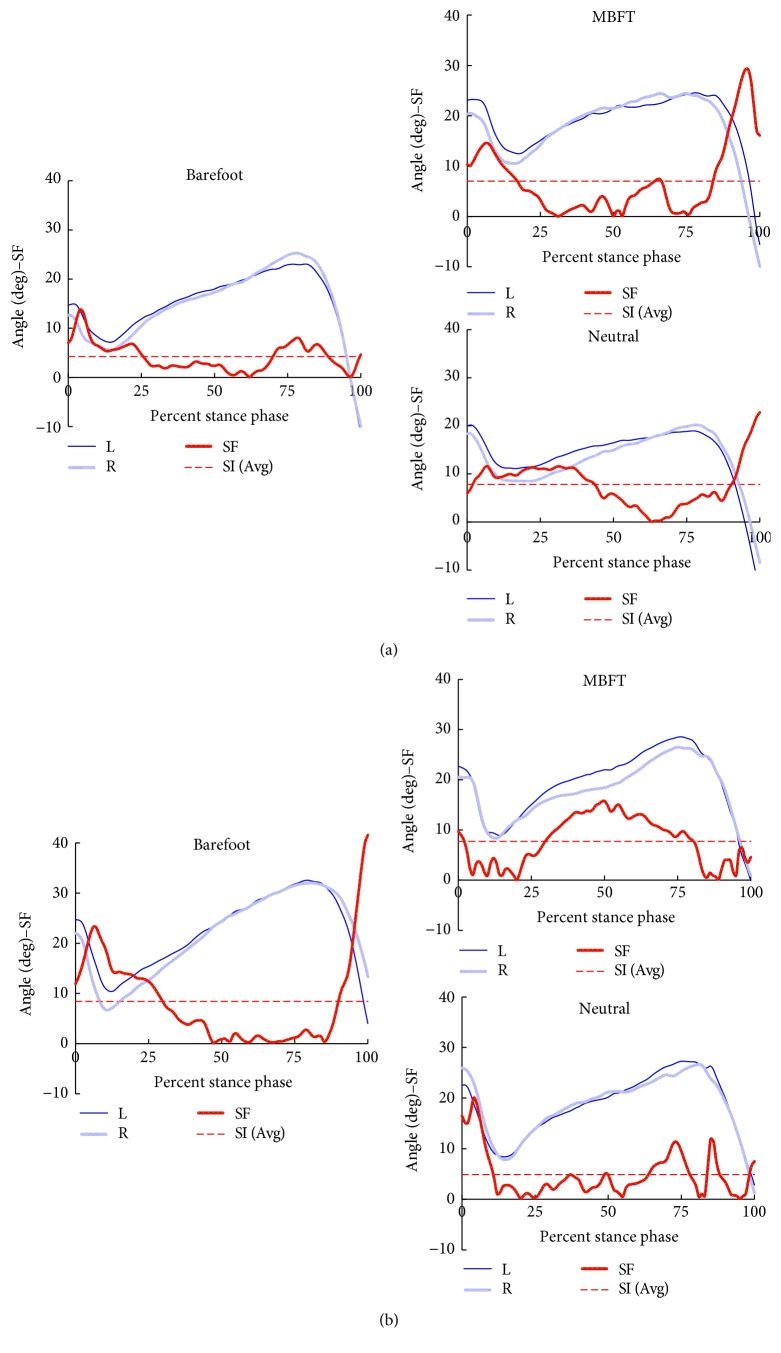
Curves illustrate the adaptive response in joint angle symmetry of subject 12 (a) and subject 13 (b). Left side (dark blue solid line), right side (light blue solid line), the curve of symmetry function (SF) was also drawn to supply information about the time dependency of symmetry during the stance phase (red dash-dot line), and the level of SI was illustrated as a dot line (red).

Judged by the value of SI, joint angle asymmetry was seen in each of the 3 motion planes and each of the 3 lower limb joints (Table 1). SIs integrated from 3 motion planes (global SI of motion planes) in each pair of lower limb joint (hip/knee/ankle) and SIs integrated from three lower limb joints (global SI of joint) also indicated that there existed asymmetry in the perspective of joint angle. Also, as indicated by the values of SI, the most significant asymmetry lied in the transverse plane among the 3 motion planes of the 3 lower limb joints. Significant differences in joint angle SI caused by footwear changing were seen only in the sagittal plane, where local gait symmetry at ankle joint and global symmetry of the 3 lower limb joints (hip-knee-ankle) were significantly lower when walked barefoot as compared to the 2 shod-walking conditions (both *p* < 0.05). In most scenarios of joint angle SI analysis, though each subject's response in joint angle symmetry is quite individualized (Figure 2), the mean SI values of walking in MBFT shoe lied between those of walking barefoot and walking in neutral shoes (Table 1).

With Nigg's SI, symmetry evaluation in the joint force perspective also showed considerable asymmetry in either local or global points of view, and the transverse motion plane has the most prominent asymmetry regarding the SI values in all of the 3 lower limb joints (Table 2). There was no significant intergroup difference found in joint force SI among the 3 tested conditions, except between the neutral shoe and barefoot condition at ankle joint in the sagittal plane; the former had lower SI of joint force than the latter (*p* = 0.036). Again, in most scenarios of joint force SI analysis, walking in MBFT shoe had moderate SI values among the 3 tested conditions (Table 2).

SI of hip joint moment in the transverse plane of neutral shoe walking showed a trend of lower than those found in barefoot walking (*p* = 0.072), while knee joint moment in transverse plane of walking in neutral shoe was significantly more symmetric than walking barefoot as regard to the SI value (*p* = 0.003). And in the frontal plane, the SI value of ankle joint moment in barefoot walking was significantly lower than that of neutral shoe walking (*p* = 0.043). No significant difference in global symmetry was found among the 3 tested conditions in the perspective of joint moment (Table 3). Similarly, judging by the values of SI, walking in MBFT shoe had intermediate joint moment symmetry among the 3 tested conditions.

## 4. Discussion

Consistent with previous studies [6, 14–17], the present study demonstrated that diverse gait asymmetries could be seen in healthy subjects. Yet, as walking mostly happens in the sagittal plane, the solution space left for the values of SIs in this plane might then get smaller and less volatile than those in the transverse and frontal planes, and it has been reported that kinematic variables in these two planes are less reliable than in the sagittal plane [18]. The relatively higher SIs in the transverse and frontal planes are then not hard to comprehend.

Previously, some authors hypothesized that gait asymmetry in healthy adults might be related to age, lateral dominance, or literalities. However, experiments designed to clarify this issue failed to find any correlation between them [15, 19, 20]. Suggesting that gait asymmetry might be intrinsic to healthy adults. Additionally, by using an optoelectronic set-up, Ferrario et al. [21] quantify the asymmetries in foot dorsi-plantar flexion, ankle range of motion, and its coupled foot movements individually in 75 young healthy and find that the percentage of subjects that have principal plane asymmetries >5 degrees is rather high (20% of female and 34% of male subjects), and subjects that have asymmetries >5 degrees in the associated movements are even higher (50%), indicating that biomechanics asymmetry might have anatomic basis. As modern society people have grown up with shoes, the difference in shoe style preference (like some who would prefer high heeled shoes) will alter the natural position of foot-ankle complex or cause a sequential anatomic alternation up to the lower limbs or even to the spine [22, 23] and may ultimately manifest as disorganization of weight-bearing distribution [24]. The results of this process can be individualized to each person and to each limb, which might then contribute to the asymmetry in gait. The individualized response in joint angle symmetry demonstrated in the present study supports this hypothesis.

Footwear has an impact on gait symmetry. In an experiment on 11 healthy adults, Aruin and Kanekar [25] notice that a textured insole put in one side of shoe can significantly modify the immediate symmetry during standing and walking. While in another observation of 15 healthy subjects performing barefoot and shod over ground running trials, Hoerzer et al. [26] notice that gait asymmetry of the participants is reduced when running in shoes as compared to running barefoot. In the present study, the kinetics calculations for force and moment perspectives are reflections of the ground reaction force and kinematics and can be affected by the use of wedges, insoles, or other devices, especially in the sagittal plane [27, 28], where lower limb joints have maximum extent of motion. In addition, ground reaction forces have large transient spikes (i.e., foot contact) and therefore the sole of the shoe helps to filter/smooth these peaks out, so small shifts in the timing of the contact peaks are reduced by the compliance of the shoe heel, which will make less variability in the ground reaction force for the shod conditions and thus a lower symmetry index. However, as investigations focusing on this topic are rare, the impact of footwear on gait symmetry needs further observations.

In the present study, when healthy youth first take on barefoot walking, they immediately achieved better symmetry in joint angle relative to the 2 shod-walking conditions. While on the other hand, they lost some symmetry in joint forces and moments. Furthermore, walking in MBFT shoes rate is intermediate in terms of gait symmetry tested among the 3 tested conditions in most scenarios. This might give us two affordances: (1) having grown up with shoes, modern society people have developed a gait style that stresses the importance of joint force/loads while wearing shoes; thus, when we change back to barefoot or barefoot mimic walking, we might have some loss in these perspectives of gait symmetry that need to be adapted to. This effect may be significant in those who already have pathologies in lower limbs; further investigations on this topic are warranted. (2) We can probably interpret this as MBFT shoes actually simulate barefoot quite well. And given that the MBFT shoes provide better joint angle symmetry than neutral shoes, and better joint force/moment symmetry than barefoot during walking, and that there have been studies indicating that MBFT shoes can decrease joint moment as compared to control shoes [29], MBFT shoes might be an ideal compromise for healthy adults, depending on user intentions.

We note several limitations for the present study. First, we have a relatively small cohort of testing subjects, and the majority of them are females; the results and conclusions thus are not generable to all the population. Second, no anthropometric measurements of plantar and joint structures were done before or after the footwear changing to further support our findings. However, as all the enrolled subjects were healthy young people, we supposed that these structural reconstructions would be mild and not measurable. Future study on special groups of people with overt anatomical asymmetry or large population-based anthropometric survey could help to further confirm our findings. Third, we did not give the tested subjects a familiarization period with the new footwear and the present observation can only be interpreted as the acute effect of changing footwear; long-term effects in gait symmetry as people change to barefoot/barefoot mimics running or walking thus need further investigations.

## 5. Conclusions

Diverse gait asymmetries can be seen in healthy youth. Modern nonhabitual barefoot adults might have to sacrifice some gait symmetry in joint force/moment if they switch to barefoot walking, though at the same time they can improve their symmetry performance in joint angle. Walking in the MBFT shoes has intermediate gait symmetry performance as compared to walking barefoot and walking in neutral running shoes and might be an ideal compromise for healthy adults, depending on user intentions.

## Figures and Tables

**Table 1:** SIs in 3 conditions in joint angle perspective.

Level	Dimension of motion	Barefoot	MBFT shoe	Neutral shoe
		Mean (SD)	Mean (SD)	Mean (SD)
Hip (H)	Sagittal (S)	5.15 (1.99)	6.40 (3.03)	6.40 (2.86)
	Transverse (T)	26.23 (17.46)	32.86 (17.60)	34.91 (20.11)
	Frontal (F)	29.91 (20.27)	29.86 (19.82)	32.76 (20.06)
	Global (S-T-F)	20.43 (18.83)	23.04 (19.32)	24.69 (20.86)
Knee (K)	Sagittal (S)	8.53 (3.49)	8.37 (3.14)	8.48 (2.60)
	Transverse (T)	41.92 (37.69)	47.87 (41.62)	48.87 (41.58)
	Frontal (F)	15.04 (9.95)	16.56 (9.83)	18.56 (12.90)
	Global (S-T-F)	21.83 (26.64)	24.27 (29.85)	25.30 (30.29)
Ankle (A)	Sagittal (S)	7.55 (4.13)	9.85 (3.35)^*∗*^	10.06 (3.84)^Δ^
	Transverse (T)	25.75 (18.65)	23.53 (17.44)	25.83 (14.44)
	Frontal (F)	28.72 (20.03)	30.70 (26.51)	27.48 (18.75)
	Global (S-T-F)	20.68 (18.38)	21.36 (20.17)	21.12 (15.79)
Global (H-K-A)	Sagittal (S)	7.07 (3.59)	8.21 (3.44)^*∗*^	8.31 (3.45)^Δ^
	Transverse (T)	31.30 (27.05)	34.76 (29.41)	36.54 (29.20)
	Frontal (F)	24.56 (18.51)	25.71 (20.74)	26.27 (18.28)
	Global (S-T-F)	20.98 (21.54)	22.89 (23.54)	23.71 (23.09)

*Note*. *∗* indicates *p* < 0.05 between MBFT shoe and barefoot; Δ indicates *p* < 0.05 between neutral shoe and barefoot.

**Table 2:** SIs in 3 conditions in joint force perspective.

Level	Dimension of motion	Barefoot	MBFT shoe	Neutral shoe
		Mean (SD)	Mean (SD)	Mean (SD)
Hip (H)	Sagittal (S)	12.55 (2.51)	12.74 (2.97)	11.99 (3.11)
	Transverse (T)	21.06 (10.54)	19.78 (7.36)	19.77 (6.58)
	Frontal (F)	5.14 (1.60)	5.76 (2.81)	5.90 (3.69)
	Global (S-T-F)	12.92 (9.05)	12.76 (7.50)	12.55 (7.37)
Knee (K)	Sagittal (S)	11.18 (3.27)	10.76 (2.57)	10.31 (2.86)
	Transverse (T)	16.53 (6.85)	14.82 (4.30)	14.93 (4.56)
	Frontal (F)	5.61 (1.57)	6.54 (3.36)	6.69 (4.32)
	Global (S-T-F)	11.11 (6.29)	10.71 (4.83)	10.64 (5.20)
Ankle (A)	Sagittal (S)	20.26 (4.78)	18.98 (3.90)	17.72 (4.63)^Δ^
	Transverse (T)	25.87 (15.85)	24.00 (14.90)	24.68 (11.69)
	Frontal (F)	5.36 (1.65)	6.29 (3.47)	6.54 (4.49)
	Global (S-T-F)	17.16 (12.88)	16.42 (11.72)	16.32 (10.70)
Global (H-K-A)	Sagittal (S)	14.66 (5.39)	14.16 (4.73)	13.34 (4.79)
	Transverse (T)	21.15 (12.16)	19.54 (10.50)	19.80 (9.02)
	Frontal (F)	5.37 (1.60)	6.20 (3.20)	6.38 (4.14)
	Global (S-T-F)	13.73 (10.07)	13.30 (8.80)	13.17 (8.39)

*Note*. Δ indicates *p* < 0.05 between neutral shoe and barefoot.

**Table 3:** SIs in 3 conditions in joint moment perspective.

Level	Dimension of motion	Barefoot	MBFT shoe	Neutral shoe
		Mean (SD)	Mean (SD)	Mean (SD)
Hip (H)	Sagittal (S)	21.77 (8.66)	22.83 (7.45)	20.81 (7.10)
	Transverse (T)	27.42 (3.36)	26.08 (3.09)	25.52 (4.94)
	Frontal (F)	27.30 (9.26)	25.71 (9.17)	24.23 (9.09)
	Global (S-T-F)	25.50 (7.94)	24.87 (7.12)	23.52 (7.43)
Knee (K)	Sagittal (S)	29.88 (15.56)	30.16 (10.52)	28.18 (10.00)
	Transverse (T)	34.03 (5.09)	31.49 (4.82)	29.62 (6.11)^Δ^
	Frontal (F)	30.24 (13.41)	28.73 (12.88)	28.35 (10.49)
	Global (S-T-F)	31.39 (12.21)	30.13 (9.94)	28.72 (9.00)
Ankle (A)	Sagittal (S)	24.50 (11.97)	23.88 (8.72)	23.61 (7.94)
	Transverse (T)	12.16 (2.50)	12.75 (4.60)	12.25 (2.50)
	Frontal (F)	40.53 (15.54)	44.42 (16.95)	49.64 (17.30)^Δ^
	Global (S-T-F)	25.73 (16.24)	27.02 (17.30)	27.08 (17.58)
Global (H-K-A)	Sagittal (S)	25.38 (12.70)	25.62 (9.45)	24.20 (8.88)
	Transverse (T)	24.54 (9.95)	23.44 (8.96)	22.46 (8.82)
	Frontal (F)	32.69 (14.05)	32.95 (15.59)	34.07 (16.90)
	Global (S-T-F)	27.54 (12.84)	27.34 (12.37)	26.91 (13.13)

*Note*. Δ indicates *p* < 0.05 between neutral shoe and barefoot.
